# Immune Checkpoint Blockade in HER2-Positive Breast Cancer: What Role in Early Disease Setting?

**DOI:** 10.3390/cancers13071655

**Published:** 2021-04-01

**Authors:** Cinzia Solinas, Debora Fumagalli, Maria Vittoria Dieci

**Affiliations:** 1 Medical Oncology, Azienda Tutela della Salute Sardegna, San Francesco Hospital, 08100 Nuoro, Italy; 2 Breast International Group, 1000 Brussels, Belgium; debora.fumagalli@gmail.com; 3 Medical Oncology 2, Veneto Institute of Oncology IOV-IRCCS, 35128 Padova, Italy; mariavittoriadieci@gmail.com

**Keywords:** HER2-positive, breast cancer, immune checkpoint blockade, immune checkpoint molecules, PD-1/PD-L1, immunotherapy

## Abstract

**Simple Summary:**

This work aims to discuss how an anti- or pro-tumor immune response could be manipulated through immune checkpoint blockade in patients with early stage HER2-positive breast cancer. By summarizing previously published evidence in the field, authors present their personal view on how immune checkpoint blockade could be implemented in the neoadjuvant setting in this patient population. The hypothesis being presented is that an appropriate and effective administration of immune checkpoint blockade could assure a lasting control of the disease, by preventing relapses. One of the research priorities should be the identification of the patients who could benefit more by this strategy.

**Abstract:**

The present commentary synthesizes the current evidence on the role of the immune response in HER2-positive breast cancer. It points out the strengths and weaknesses of the findings observed so far, particularly in the early setting, including the clinical significance of scoring tumor-infiltrating lymphocytes. A figure proposing research hypotheses for the implementation of immune checkpoint blockade use for patient candidates to neoadjuvant treatment is presented.

## 1. Introduction

Immune checkpoint blockade represents a successful immunotherapy strategy aimed at boosting a pre-existing adaptive anti-tumor immune response with a potential lasting control of the disease [1]. The latter could be achieved because adaptive immunity is characterized by memory and by the possibility to simultaneously target the tumor-associated antigens that can be generated as cancer develops. This innovative treatment approach is revolutionary for the durable responses it can induce. Further, stimulating the immune system could potentially target multiple neo-antigens over time. All these peculiar aspects have generated a great interest in identifying the patient subsets that benefit more from immune checkpoint blockade, particularly in early settings, where the chances of cure are higher for cancer patients.

After the introduction of effective anti-human epidermal growth factor receptor-2 (HER2) agents, early-stage HER2-positive breast cancer patients have experienced improved survival [2]. Some of these patients achieve excellent outcomes and are ideal candidates for de-escalation treatment strategies aimed at sparing toxicities from chemotherapy, as explored in studies like CompassHER2-pCR (NCT04266249) and Decrescendo (NCT04675827) [3]. However, up to 20% of early stage HER2-positive breast cancer patients still relapse and develop an advanced disease [2]. For these patients, innovative add-on strategies should be developed. Upfront use of immune checkpoint blockade could be useful in this context, but it is not known which patients could benefit more from this approach.

The aim of this commentary is to briefly summarize evidence on the relationship between a spontaneous immune response (i.e., the one present in inflamed tumors that are characterized by a detectable immune infiltration) and outcome and/or responses to standard treatments in HER2-positive breast cancer. In addition, some research hypotheses for the experimental use of immune checkpoint blockade in the neoadjuvant setting are proposed, which take into account the implementation of immune-related biomarkers and add-on or de-escalation treatment strategies. Further, research priorities are identified.

## 2. HER2-Positive Breast Cancer Patients and Tumor-Infiltrating Lymphocytes: Clinical Significance

The HER2-positive subtype represents up to 20% of breast cancer diagnoses [4]. Its main oncogenic driver is represented by the amplification and over expression of HER2, whose activity is inhibited by a variety of anti-HER2 agents that are nowadays being used in the clinic, with relevant clinical benefit in all the settings of the disease [2]. This subtype is characterized by heterogeneity, mostly driven by the presence or absence of the hormone receptors’ expression, which identifies distinct subgroups with different prognoses and responses to treatments [5].

Gene-expression data reveal that up to 50–60% of HER2-positive breast cancers have a HER2-enriched PAM50 molecular profile, particularly hormone receptor-negative (75%) compared to hormone receptor-positive (30%) cancers [6,7,8,9]. This molecular subtype is characterized by high activity in HER2 signaling, rendering these tumors HER2 hypersensitive, thus particularly responsive to anti-HER2 targeted agents [10]. Other predictors of benefit from these drugs are: high expression of the *ERBB2* gene [10] and of immune gene signatures [11], the latter being able to predict a higher likelihood of pathological complete response after neoadjuvant treatments (an intermediate endpoint for survival [12]), and a longer event-free survival [13]. In addition, a high extent of tumor-infiltrating lymphocytes (TIL) evaluated on hematoxylin and eosin (HE)-stained slides has been associated with better outcomes (pathological complete response, event-free survival and disease-free survival) in several trials, confirming the relevant role played by the host immunity during the course of this disease [14,15].

TIL represent a well recognized biomarker in the cancer field, particularly in this new era of cancer immunotherapy. TIL are easily, reproducibly and cheaply scored by pathologists on HE slides used in clinical routine [16]. In the HER2-positive breast cancer subtype, higher TIL levels were observed in the hormone receptor-negative with respect to the hormone receptor-positive subgroup [17,18].

Immunohistochemical (IHC) analysis allows to identify the various immune cell subsets that are globally scored as TIL on HE slides (based on morphological features). These include: Helper T cells (Th, CD4^+^), cytotoxic T cells (CTL, CD8^+^), B cells (CD20^+^) from the adaptive immunity; macrophages, neutrophils, myeloid derived suppressor cells, natural killer (NK) from the innate immunity [19]. Among the cells of the adaptive immunity (characterized by immunological memory), Th lymphocytes contribute to the development of a response by activating tumor antigen-specific effector lymphocytes (CTL) and through the recruitment of various cells of the innate immunity. There are several subpopulations of Th, such as the anti-tumor Th1 (able to kill tumor cells by releasing cytokines that activate death receptors on target cells, and to produce cytokines that activate CTL), and the Th2 that promote a pro-tumor microenvironment. CTL kill tumor cells, and B cells are able to secrete antibodies and cytokines, assuring an effective anti-tumor immune response in breast cancer [20].

Of interest, the prognostic relevance of TIL scored on HE slides in neoadjuvant and adjuvant trials of HER2-positive breast cancer has been shown to be independent both of other clinicopathological characteristics [21,22] and of the administered treatment [15]. The various anti-HER2 compounds exert in fact different immune effects [5] through complex interactions between the various cells of the tumor microenvironment (particularly those from innate immunity) and the treatments (including chemotherapy) co-administered. Specifically, (1) the anti-HER2 monoclonal antibody trastuzumab increases the antibody-dependent cell-mediated cytotoxicity (ADCC) since its Fc portion (the tail region of an antibody that does not recognize the antigen) interacts with the Fcγ receptors expressed on the effector cells of the innate immunity (NK cells, neutrophils and γδT-cells) and with some proteins of the complement system [5]. Similar effects are seen with (2) the anti-HER2 monoclonal antibody pertuzumab that induces ADCC and augments the density of Fcγ receptor binding sites on HER2-positive cells, potentiating NK cell activity [5], and with (3) the antibody drug conjugate trastuzumab emtasine (T-DM1), which increases ADCC [5]. The (4) tyrosine kinase inhibitor lapatinib raises the density of HER2 receptors on the surface of tumor cells, potentiating the ADCC associated with trastuzumab [5]. Anthracyclines, taxanes, cyclophosphamide and other cytotoxic agents employed in breast cancer can also mediate immune effects [23].

Thus, it is expected that stratification of patients on the basis of the level of immune infiltration of their tumors (reflected by the extent of baseline TIL on HE slides) should allow to identify patients that are more likely to benefit from standard treatments and/or experience better outcomes. Further, it could be helpful in identifying those patients at higher risk of relapse (those having lower TIL) who might have the opportunity to be treated with immune checkpoint blockade as an add-on strategy.

## 3. Potential Role of Immune Checkpoint Blockade in HER2-Positive Breast Cancer

A growing interest in manipulating the patient’s own immune response against cancer has followed the introduction of immune checkpoint blockade strategies. One of the crucial steps for further exploration of this treatment strategy is represented by the identification of the target patient population.

Evidence from studies in triple negative breast cancer, the breast cancer subtype showing the highest levels of TIL infiltration, reveals that programmed cell death-1 or its ligand (PD-(L)1) immune checkpoint blockade is more active in early settings, independent of PD-L1 expression on tumor cells, lymphocytes and macrophages [24]. In contrast, in the advanced disease the presence of PD-L1 expression by immune cells identifies the subgroup that benefits more from the association of an anti-PD-L1 plus chemotherapy [25].

In HER2-positive breast cancer limited efficacy of immune checkpoint blockade combined with T-DM1 or trastuzumab has been observed in the advanced setting in patients previously treated with trastuzumab and expressing PD-L1 on immune cells and/or having higher levels of TIL [26,27]. However, these results derive from hypothesis-generating trials and deserve further confirmation, also considering that early and late diseases appear to have a different biological profile. For example, the levels of TIL on metastatic HER2-positive samples may be lower than matched primary tumors [28] and the infiltration by CTL may be lower in metastatic samples from patients pre-treated with chemotherapy and anti-HER2 therapy [29].

So far, not much is known about the role of immune checkpoint blockade in early settings in this subtype, also considering the variety and efficacy of the anti-HER2 drugs now available (i.e., trastuzumab +/− pertuzumab as part of the (neo)adjuvant treatment and T-DM1 as part of the post surgical treatment in patients with a residual disease after neoadjuvant treatment).

Biologically, a mouse model revealed that the efficacy of immune checkpoint blockade in early settings was higher in the presence of tumor antigens (i.e., as in the case of a neoadjuvant approach), rather than in their absence [30]. Ideally, administration of immune checkpoint blockade would be more efficacious as part of the upfront neoadjuvant treatment space, which in HER2-positive breast cancer currently looks crowded with several treatment strategies being explored [31].

However, some studies have shown that tumors with high TIL (the most infiltrated that traditionally benefit most from immune checkpoint blockade) are also characterized by excellent responses to neoadjuvant treatments with anti-HER2 agents plus taxane +/− anthracycline-based chemotherapy regimens and by excellent prognosis (event-free survival) [15,17].

Whether patients with highly infiltrated tumors really need a boost to their immunity with immune checkpoint blockade, considering that standard treatments are very efficacious, is still a crucial point that needs to be addressed.

## 4. Potential Use of Immune Checkpoint Blockade in the Neoadjuvant Setting for HER2-Positive Breast Cancer Patients

**Figure 1:**
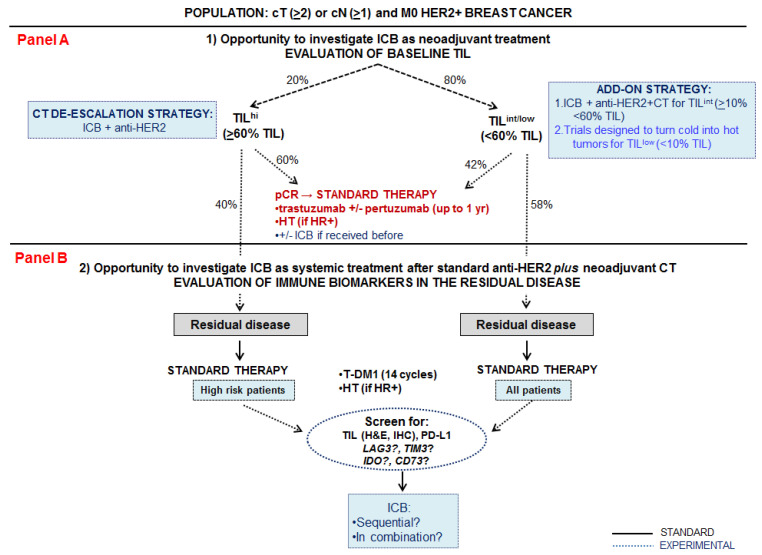
Figure 1 represents proposed research hypotheses for the use of immune checkpoint blockade (ICB) in early-stage HER2-positive breast cancer patients who are candidates for neoadjuvant therapy (≥cT2 or ≥cN1 and M0) with the employment of tumor-infiltrating lymphocytes (TIL) assessed on diagnostic biopsies as an additional biomarker to guide treatment decisions. Panel (**A**) shows upfront treatment strategies whereas panel (**B**) proposes the treatment strategies to be administered in the presence of a residual disease after neoadjuvant therapy (panel (**B**)). References: [15,34]. Legend: CT, chemotherapy; HE, hematoxylin and eosin; HR, hormone receptor; HT, hormone therapy; ICB, immune checkpoint blockade; IHC, immunohistochemistry; M, metastasis; N, nodal status; pCR, pathologic complete response; T, tumor size; T-DM1, trastuzumab emtansine; TIL, tumor-infiltrating lymphocytes; TIL^hi^, ≥60% tumor-infiltrating lymphocytes; TIL^int^, ≥10% <60% tumor-infiltrating lymphocytes; TIL^lo^, <10% tumor-infiltrating lymphocytes.

The neoadjuvant setting represents an ideal scenario to test drug sensitivity, characterize the biology of the disease, identify reliable predictive biomarkers and to guide treatment decisions based on the presence of a residual disease [32]. Current guidelines suggest that neoadjuvant treatment in HER2-positive breast cancer patients should be administered for clinical ≥ T2 or ≥N1 (Figure 1) [33]. Figure 1 summarizes various research hypotheses that could be tested upfront (panel A) or in the presence of a residual disease after neoadjuvant therapy (panel B) in HER2-positive breast cancer patients stratified based on the extent of baseline TIL assessed on HE slides from pre-treatment biopsies. Of note, frequencies of pathological complete response achievement based on baseline TIL levels refer to HER2-positive breast cancer patients treated with trastuzumab plus anthracyclines and taxanes in the pre-pertuzumab era [15].

Baseline high TIL infiltration (with a TIL cut-off of ≥60% from previously published randomized controlled trials [15]) is found in around 20% of patients with early stage HER2-positive breast cancers and has been associated with a higher likelihood of pathological complete response (Figure 1, left side). Previous studies showed in fact that up to 60% of these patients achieve a pathological complete response (Figure 1) [15]. The remaining 40% present a residual disease, and become candidates to receive standard T-DM1 [25] +/− hormone therapy (according to the baseline hormone receptors status). In this patient population, upfront anti-PD-(L)1-based immune checkpoint blockade could be administered in association with dual anti-HER2 blockade (trastuzumab plus pertuzumab) as a chemotherapy de-escalation strategy (Figure 1, panel A, left side).

The remaining 80% of HER2-positive breast cancer patients treated in the neoadjuvant setting (Figure 1, right side) represent a highly heterogeneous group including tumors with either a very low (TIL^lo^ usually below 10%) or with an intermediate TIL (TIL^int^ ranging from 10% up to 60%) infiltration. Around half of these patients achieve a pathological complete response after standard anti-HER2 therapy, whereas the other half have a residual disease [15] and will be candidates for T-DM1 +/− hormonotherapy. Upfront anti-PD-(L)1-based immune checkpoint blockade could be associated with chemotherapy and standard anti-HER2 treatments (pertuzumab plus trastuzumab), particularly in the group of patients with baseline intermediate TIL, as an add-on strategy (Figure 1, panel A, right side). The group of patients with low TIL should theoretically be ideal candidates for strategies aimed at increasing immune infiltration, such as vaccines [35] or adoptive T cell transfer [36] though these treatments need further investigations in breast cancer.

Patients from both groups (Figure 1, panel A, left and right side) who have achieved a pathological complete response could be candidates to receive immune checkpoint blockade plus standard anti-HER2 treatment if immunotherapy was previously administered (Figure 1, panel A).

## 5. Potential Use of Immune Checkpoint Blockade in the Presence of a Residual Disease after Neoadjuvant Therapy

Even though HER2-positive breast cancer patients with baseline high TIL achieve a better event-free survival [17], it is not clear whether this also applies to patients who have a residual disease after neoadjuvant therapy.

For patients with a residual disease after standard neoadjuvant treatment (chemotherapy plus anti-HER2 agents), nodal involvement and estrogen receptor negativity have been associated with shorter event-free survival [37]. The presence of high TIL (≥25%) in the residual disease was associated with worse event-free survival in a retrospective study, questioning whether in this context the use of immune checkpoint blockade might have a role in rescuing the activity of potentially dysfunctional immune cells [34]. Interestingly, the expression of Immunoglobulin (Ig)-G in the residual disease was associated with longer event-free survival [13], signifying that the presence of a well-organized adaptive immunity, including B (that produce Ig) and T lymphocytes, assures a lasting control of the disease. Hence, this highlights the need to identify the subsets of immune cells that constitute the immune infiltrate (i.e., Th, CTL, B lymphocytes, macrophages, etc.), in addition to scoring them only on HE slides.

Further, the presence of a residual disease might represent an opportunity to screen patients for anti PD-(L)1-based combinations of immune checkpoint blockade through the evaluation of the expression of PD-L1, and of other inhibitory immune checkpoint molecules, such as: Lymphocyte activation gene 3 (LAG3), T cell immunoglobulin and mucin domain 3 (TIM3) which are heterogeneously expressed in early-stage HER2-positive breast cancer [38,39] and which tightly regulate the immune response through inhibitory, non-redundant pathways.

Patients at high risk of relapse for clinicopathological factors, but with baseline high TIL and a residual disease after neoadjuvant therapy (Figure 1, panel B, left side) could be eligible to receive a rescue treatment with immune checkpoint blockade, after assessment of various immune biomarkers on the surgical specimen.

Concerning those patients with baseline intermediate TIL and a residual disease after neoadjuvant therapy (Figure 1, panel B, right side) they could all be evaluated for TIL and other immune biomarkers on the surgical specimen in order to characterize their eligibility for an immune checkpoint blockade, as an add-on treatment strategy besides standard treatments.

Whether this strategy should be given sequentially or in concomitance with standard treatments in patients with a residual disease might need to be addressed by dedicated studies.

- Additional multiple immune biomarkers

Beside TIL, PD-L1, LAG3 and TIM3, evaluation of other markers in the residual disease, such as CD73 and IDO, might be important, particularly in patients previously treated with PD-(L)1 blockade (i.e., administered as an upfront strategy) [40,41] (Figure 1, panel B).

So far, PD-L1 expression by IHC is performed as a biomarker for patient selection in trials of anti-PD-1/PD-L1-based immune checkpoint blockade. This also applies to LAG3 expression, which was shown to be associated with an improved benefit from PD-1 plus anti-LAG3 combinations [42]. Evaluation of the expression of multiple immune checkpoint molecules (starting from those for which drug compounds have been developed) might be achieved with the use of multiplex-IHC, a technique that allows the identification of several markers in one tissue-stain, or with the use of in situ hybridization techniques allowing the localization of the expression of genes, at both mRNA and protein levels within a histological section of a single tissue or tissue microarrays [43]. Cut-off values for positivity (usually set at >1% of positive cells, as for PD-L1 assessment) and standardization of the techniques need to be confirmed and validated.

Indeed, apart from PD-(L)1, it is not known which of these molecules is the most promising in breast cancer, considering that they can be heterogeneously expressed in tumors with intermediate or high TIL [38]. Thus, an entire panel of immune checkpoint molecules and other markers should be analyzed, also considering that they regulate non-redundant inhibitory pathways. Remarkably, by flow cytometry PD-1 and CTLA-4 are usually always expressed in inflamed breast tumors, whereas LAG3 and TIM3 are rarely highly expressed at the protein level in untreated early-stage breast tumors [38]. Ideally, the identification of different markers might represent a guide indicating which inhibitory pathway should be worthwhile as a target for immune checkpoint blockade in each patient.

## 6. Research Priorities in the Immunotherapy Field

To support the implementation of immune blockade strategies, some aspects need to be further investigated:

(1) The ideal cut-off for TIL in the different settings (i.e., adjuvant, neoadjuvant, at baseline and in the residual disease), considering the wide variety of thresholds used in the literature.
(2) The ideal cut-off (if any) for PD-L1 in early settings when considering the lesson learnt from neoadjuvant trials in triple negative breast cancer, revealing that the benefit from immune checkpoint blockade is remarkable regardless of PD-L1 status.
(3) Concerning PD-L1 assessment: should it be done on immune cells only (as previously performed in advanced HER2-positive breast cancer) or in both immune and tumor cells (as it is through the combined positive score in early-stage triple negative breast cancer)?
(4) The need to explore strategies that are alternatives to standard treatments (anti-HER2 agents and chemotherapy) with the aim of enhancing the immune response in hot (high TIL) tumors and convert cold (low TIL) into hot tumors in the early setting.
(5) The potential use of immune checkpoint blockade in chemotherapy de-escalating strategies (i.e., in already inflamed, high TIL tumors) in the early setting.
(6) The potential use of immune checkpoint blockade in escalation treatment strategies for early-stage HER2-positive low TIL breast tumors.
(7) The difference between the hormone receptor-positive (less immunogenic) and hormone receptor-negative (more immunogenic) subgroups in HER2-positive breast cancer with regards to sensitivity to immune checkpoint blockade.
(8) Identification of reliable novel immune (and not) biomarkers for patient selection.

Future studies will hopefully help address these research priorities.

## 7. Conclusions

The immune revolution challenges researchers in various ways, and the selection of patients that most likely derive lasting benefits from novel treatments represents one of the most important aspects. Diverse biomarkers have been proposed, and, among them, TIL represent the most standardized and cheaply assessed immune-related biomarker so far employed in breast cancer. However, future approaches will benefit from a more in depth characterization of the tumor microenvironment, including evaluation of the composition of TIL, of their functional profiles, of the expression of immune checkpoint molecules and from answers to open research questions as summarized above.

With this commentary, we provide an overview of the current knowledge in the field and propose an opportunity for exploitation of immune checkpoint blockade to further improve the outcome of patients with HER2-positive breast cancer.

## Data Availability

Not applicable.
